# 572. Growth rate comparisons for clinical *Klebsiella pneumoniae* (KP) isolates using nutrient-rich and -depleted broth stratified by inner colony production during fosfomycin (FOF) disk diffusion (DD) testing

**DOI:** 10.1093/ofid/ofad500.641

**Published:** 2023-11-27

**Authors:** Ellora Daley, Morgan Bixby, Lindsey Collins, Jenna Salay, Elizabeth B Hirsch

**Affiliations:** University of Minnesota College of Pharmacy, Minneapolis, Minnesota; University of Minnesota College of Pharmacy, Minneapolis, Minnesota; University of Minnesota College of Pharmacy, Minneapolis, Minnesota; University of Minnesota College of Pharmacy, Minneapolis, Minnesota; University of Minnesota College of Pharmacy, Minneapolis, Minnesota

## Abstract

**Background:**

Recent studies have indicated the presence of non-susceptible IC appearing within the zone of inhibition during FOF DD testing. Limited studies focusing on *Escherichia coli* have demonstrated that observed increases in resistance come with decreased isolate fitness. We sought to compare growth rates of susceptible KP non-IC-producers, IC-producers, and their non-susceptible IC (as determined by broth microdilution) in both nutrient-rich and -depleted broth to understand *in vitro* fitness differences.

**Methods:**

Two isolates which had never produced IC (254, 275), two susceptible IC-producers (131, 209), and two corresponding resistant IC (131D, 209D) were included. Each isolate was grown in both full and ¼ strength Mueller-Hinton broth (MHB) for 24 hours with samples taken at standard time intervals in two technical replicates, with duplicates performed on separate days. Using the generation time equation (Figure 1) for the logarithmic phase of growth, rates were calculated and compared across three groups: (1) individual parent and IC pairs, (2) non-IC producers and IC-producers, and (3) IC-producers and IC as a whole. Comparisons across the three groups in both nutrient-rich and -depleted conditions were completed using Student’s t-tests.

**Figure:**
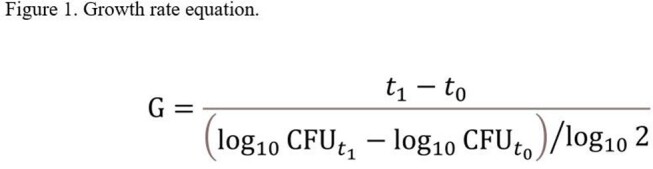

**Results:**

Average generation time for each isolate in full and ¼ strength MHB are shown in Table 1. Difference in generation time in full-strength MHB for parent and IC pairs were [131/131D] 0.04 hours and [209/209D] 0.05 hours (Table 2). In ¼ strength MHB, difference in generation time for parent and IC pairs were [131/131D] 0.03 hours and [209/209D] 0.01 hours. Average growth rate difference between non-IC-producers and IC-producers was 0.05 hours and 0.01 in full- and ¼ strength, respectively. The average generation time difference between IC-producers and IC was < 0.01 hours and 0.01 in full- and ¼ strength, respectively.

**Figure:**
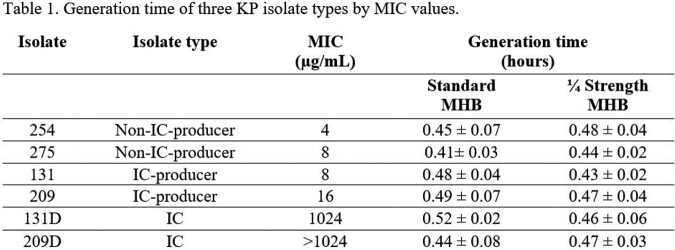

**Figure:**
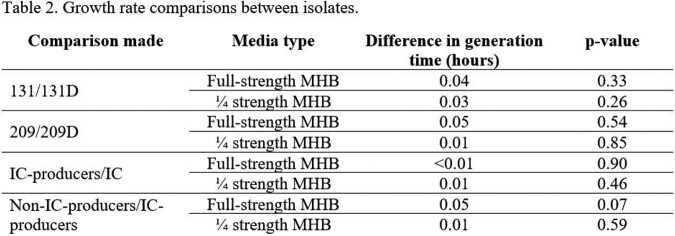

**Conclusion:**

Our data reveal no significant difference in growth rates between these KP isolate types which may signify no *in vitro* fitness differences for IC or IC-producers in both nutrient-rich and nutrient-depleted conditions. While the findings of this study are limited due to small sample size, they justify further investigation into the fitness of these isolate types through an *in vivo* mouse model and genetic testing.

**Disclosures:**

**Elizabeth B. Hirsch, PharmD, FCCP, FIDSA**, Melinta: Honoraria|Merck: Grant/Research Support

